# Bersavine: A Novel Bisbenzylisoquinoline Alkaloid with Cytotoxic, Antiproliferative and Apoptosis-Inducing Effects on Human Leukemic Cells

**DOI:** 10.3390/molecules25040964

**Published:** 2020-02-20

**Authors:** Darja Koutova, Monika Kulhava, Radim Havelek, Martina Majorosova, Karel Královec, Klara Habartova, Anna Hošťálková, Lubomír Opletal, Lucie Cahlikova, Martina Řezáčová

**Affiliations:** 1 Department of Medical Biochemistry, Faculty of Medicine in Hradec Kralove, Charles University, Simkova 870, 500 03 Hradec Kralove, Czech Republic; koutova.darja@lfhk.cuni.cz (D.K.); pospismo@lfhk.cuni.cz (M.K.); SeifrtovaM@lfhk.cuni.cz (M.M.); habartova.klara@gmail.com (K.H.); rezacovaM@lfhk.cuni.cz (M.Ř.); 2 Department of Biological and Biochemical Sciences, Faculty of Chemical Technology, University of Pardubice, Studentska 573, 532 10 Pardubice, Czech Republic; Karel.Kralovec@upce.cz; 3 ADINACO Research Group, Department of Pharmaceutical Botany, Faculty of Pharmacy, Charles University, Heyrovskeho 1203, 500 05 Hradec Kralove, Czech Republic; HOSTA4AA@faf.cuni.cz (A.H.); opletal@faf.cuni.cz (L.O.); cahlikova@faf.cuni.cz (L.C.)

**Keywords:** bersavine, antiproliferative activity, cytotoxicity, cell cycle, apoptosis

## Abstract

Bersavine is the new bisbenzylisoquinoline alkaloid isolated from the *Berberis vulgaris* L. (Berberidaceae) plant. The results of cytotoxicity screening 48 h post-treatment showed that bersavine considerably inhibits the proliferation and viability of leukemic (Jurkat, MOLT-4), colon (HT-29), cervix (HeLa) and breast (MCF-7) cancer cells with IC_50_ values ranging from 8.1 to 11 µM. The viability and proliferation of leukemic Jurkat and MOLT-4 cells were decreased after bersavine treatment in a time- and dose-dependent manner. Bersavine manifested concentration-dependent antiproliferative activity in human lung, breast, ovarian and hepatocellular carcinoma cell lines using a xCELLigence assay. Significantly higher percentages of MOLT-4 cells exposed to bersavine at 20 µM for 24 h were arrested in the G1 phase of the cell cycle using the flow cytometry method. The higher percentage of apoptotic cells was measured after 24 h of bersavine treatment. The upregulation of p53 phosphorylated on Ser392 was detected during the progression of MOLT-4 cell apoptosis. Mechanistically, bersavine-induced apoptosis is an effect of increased activity of caspases, while reduced proliferation seems dependent on increased Chk1 Ser345 phosphorylation and decreased Rb Ser807/811 phosphorylation in human leukemic cells.

## 1. Introduction

Over the past decades, drugs derived from plants have continued to have beneficial health effects in the prevention and treatment of many diseases. Natural products and plants have made an enormous impact on the discovery of anticancer drugs. Approximately 60% of all cancer drugs that are used clinically are either natural products, analogues of the parent natural products, or mimics [1]. Although plants have been identified as a sound source of anticancer agents, the promise of combinational chemistry and modern synthetic technologies has overshadowed natural product research as a source of new drugs [2]. Plants historically stand at the core of medicine, and they are still a major source of prospective new drug leads. Although plants represent a superb source of the most effective anticancer drugs, such as vinca alkaloids, taxanes, podophyllotoxin derivatives and others, a large number of plant-derived compounds have barely been studied and still need to be investigated [3].

**Figure 1:**
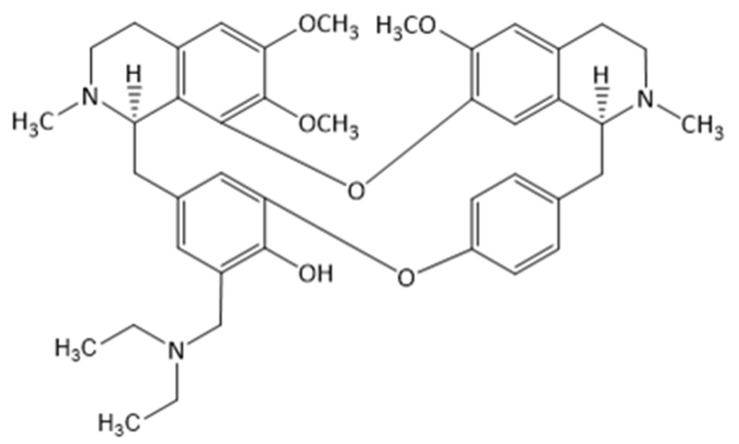
The chemical structure of bersavine.

The Berberidaceae family is a rich source of isoquinoline alkaloids of various structural types, including bisbenzylisoquinoline alkaloids, which are characterized by having the benzylisoquinoline skeleton dimerized by ether linkages or carbon-carbon bonds. Their diversity is due to the different substituents on the aromatic rings. They are biosynthetically obtained from benzyltetrahydroisoquinoline units through phenolic oxidative coupling [4]. The representative of this structural type is alkaloid berbamine from *Berberis vulgaris* [5]. Since berbamine showed exquisite potency against cancer cell lines, there is a dire need to investigate and bring to light the evidence for possible anticancer activity of hitherto unexplored novel bisbenzylisoquinoline alkaloid bersavine (Figure 1).

Bersavine, a natural bisbenzylisoquinoline alkaloid, has been recently isolated for the first time from the root bark *Berberis vulgaris* L. (Berberidaceae) [5]. Bersavine, in structural analogy with berbamine, incorporates two tetrahydrobenzylisoquinoline moieties combined through two ether linkages. Berbamine has been known for its promising cytotoxic activity on human cancer cell lines in vitro for a long time, and it has also been shown to stimulate normal hematopoiesis and enhance immune function in cancer patients [6]. Berbamine also exerts anti-inflammatory effects by inhibiting nuclear factor-kappaB (NF-κB) and mitogen-activated protein kinase (MAPK) signalling pathways [7]. Its ability to inhibit proliferation in cancer cells was described repetitively in many experimental studies [8,9,10,11,12,13]. Zhang and coworkers observed the apoptosis-inducing effect of berbamine in colorectal cancer cells by activating p53-dependent apoptotic signalling pathways [14]. Additionally, another recent study showed enhanced antimetastatic and antitumorigenic efficacy of berbamine-loaded lipid nanoparticles in vivo [15]. In the study of Jia et al., berbamine and paclitaxel were tested for their synergistic antitumor effects via the reactive oxygen species ROS/Akt pathway in glioma cells; berbamine has been shown to be a promising adjuvant to conventional chemotherapy of malignant glioma [7]. 

While berbamine has been shown to possess multiple biological activities, only one study on bersavine bioactivity has been published so far. Hošťálková et al. described the neuroprotective activity of bersavine, including the capability to inhibit human erythrocyte acetylcholinesterase (*h*AChE), human serum butyrylcholinesterase (*h*BuChE) and prolyl oligopeptidase (POP; E.C. 3.4.21.26) activities [5]. However, as far as we know, no evidence supporting the antiproliferative, cytotoxic and proapoptotic activity of bersavine has yet been described. In this study, we aimed to elucidate the antiproliferative and cytotoxic activity of bersavine in mini panels with cancer cells. To comprehensively reveal the anticancer potential of bersavine, the evidence for an apoptosis-mediated cell death, impairment in cell cycle progression, including the underpinning molecular mechanisms involved, were also investigated in human leukemic cells. 

## 2. Results

### 2.1. The Cytotoxicity of Bersavine Against Different Cancer Cell Lines

First of all, the cytotoxic effect of bersavine and berbamine were evaluated using a panel of human cancer cells, including cell lines from solid tumours (A549, HT-29, PANC-1, A2780, HeLa, MCF-7, SAOS-2), and blood cancers (Jurkat, MOLT-4) in a single-dose exposure at 10 μM, using the cell proliferation and viability water-soluble tetrazolium salt WST-1 reagent. Sensitivity to the antiproliferative activities of bersavine and berbamine following a single-dose exposure at a concentration of 10 µM was calculated as a mean growth percent (GP) value for each cell line. As shown in Table 1, the different cancer cell lines showed varied sensitivities to 48 h exposure to bersavine and berbamine. The alkaloid bersavine exhibited antiproliferative activity with a mean GP value of 66% with maximum sensitivity against MOLT-4, HeLa and MCF-7 cells. Alkaloid berbamine had a mean GP value of 74%, displaying the highest activity against HeLa, MCF-7 and Jurkat cells. As a positive control, we followed the antiproliferative effect of doxorubicin, a conventional cytostatic drug, at 1 µM, which showed a mean GP value of 37% (Table 2). Among all of the cell lines tested, Jurkat, MOLT-4, HT-29, HeLa, and MCF-7 showed the highest overall sensitivity to 10 µM of bersavine or berbamine, with a GP value under 50% of control proliferation. Thus, in the next set of experiments, the IC_50_ values of bersavine and berbamine were determined for the most sensitive cancer cell lines (cell lines demonstrating at least 50% inhibition of proliferation at 10 µM single-dose treatment) with a range from 6.1 to 16.4 µM for berbamine and 8.1 to 11.0 μM for bersavine (Table 3). Growth curves for IC_50_ value determinations are in the Supplementary Material as Figure S1.

**Figure 2:**
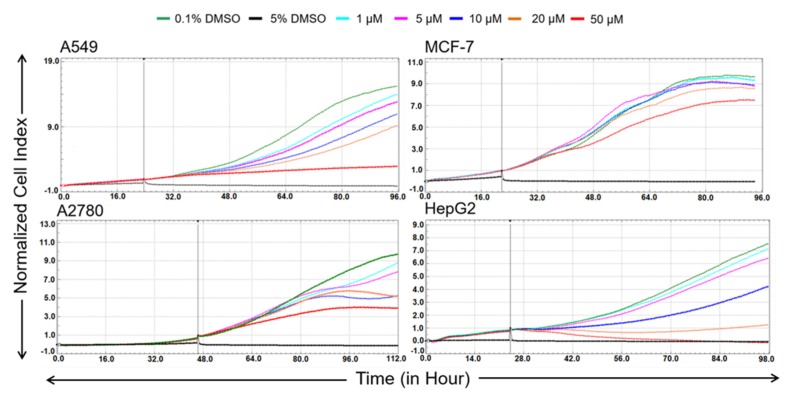
Dynamic real-time monitoring of proliferation and cytotoxicity using the xCELLigence system dedicated to adherent cell lines. Growth kinetics of human A549 lung carcinoma, A2780 ovarian carcinoma, MCF-7 breast adenocarcinoma and HepG2 hepatocellular carcinoma cells treated with bersavine. The black vertical line indicates the time point when the tested alkaloid was added. Cells treated with 0.1% DMSO were used as vehicle controls and 5% DMSO-treated cells were used as a positive control. The normalized cell index was measured over 72 h. The plots shown are representative of at least three replicate experiments in each case.

To derive further evidence of antiproliferative activity, real-time monitoring of cell growth was measured over 72 h after the application of bersavine in a range from 1 to 50 μM using the xCELLigence system in A549, A2780, MCF-7 and HepG2 cell lines. The xCELLigence system allows for the continuous quantitative monitoring of cellular behaviour, including cell adhesion, proliferation and cytotoxicity. The negative control cells were exposed to 0.1% dimethyl sulfoxide (DMSO) as a vehicle, and the positive control cells were exposed to 5% DMSO. The curvature of the xCELLigence real-time cell proliferation data indicated that bersavine (Figure 2) impacted cell proliferation, demonstrating an overall great potency towards A549 and HepG2 cells under the tested concentrations. In terms of sensitivity, Jurkat and MOLT-4 leukemic cells were selected as model cells in the following experiments.

### 2.2. The Effects of Bersavine on the Proliferation and Viability of Jurkat and MOLT-4 Cells

**Figure 3:**
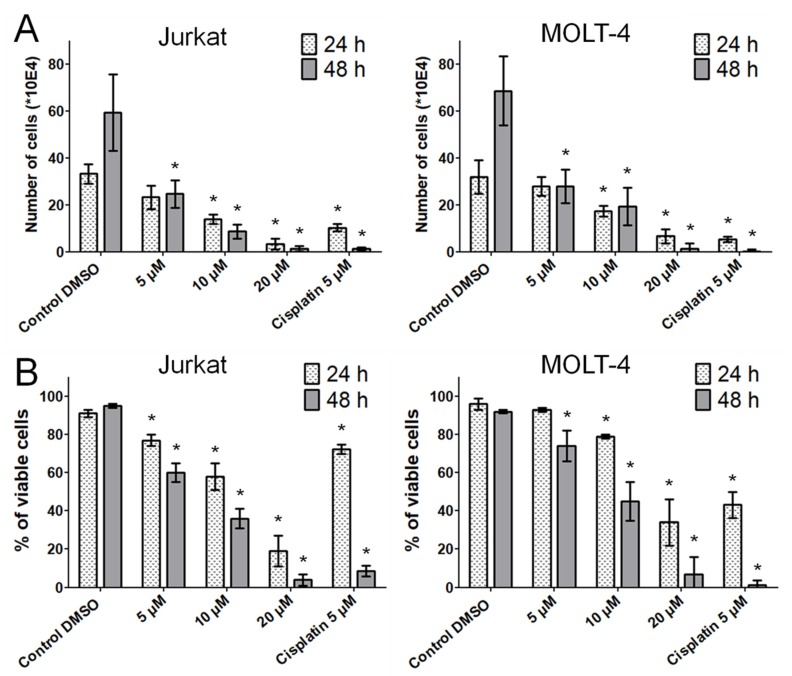
The effect of bersavine on the proliferation and viability of Jurkat and MOLT-4 leukemic cells. Changes in proliferation (**A**) and viability (**B**) were monitored for 24 and 48 h of treatment by Trypan blue exclusion analysis. Results are shown as the mean ± SD from Table 1. Cells treated with 5 µM of cisplatin were used as a positive control. * Significantly different to control (*p* ≤ 0.05).

Next, we performed a Trypan blue dye exclusion test to determine the proliferation and viability of the cells by counting the viable cells that excluded the vital stain. The significant antiproliferative effect of bersavine was confirmed at a concentration of 5 μM in Jurkat cells and MOLT-4 cells after 48 h of treatment. The antiproliferative effect of bersavine was considerably higher in Jurkat cells than in MOLT-4 cells (Figure 3A). Moreover, the results of the Trypan blue assays showed that bersavine interfered with Jurkat and MOLT-4 cell viability in a dose- and time-dependent manner. A significant reduction of viability percentage was observed in Jurkat cells at 5 μM following a 24 h interval of treatment with bersavine, but the same effect was observed in MOLT-4 cells at the same concentration after a longer period of time (Figure 3B).

### 2.3. The Effects of Bersavine on Cell Cycle Progression in Jurkat and MOLT-4 Cells

**Figure 4:**
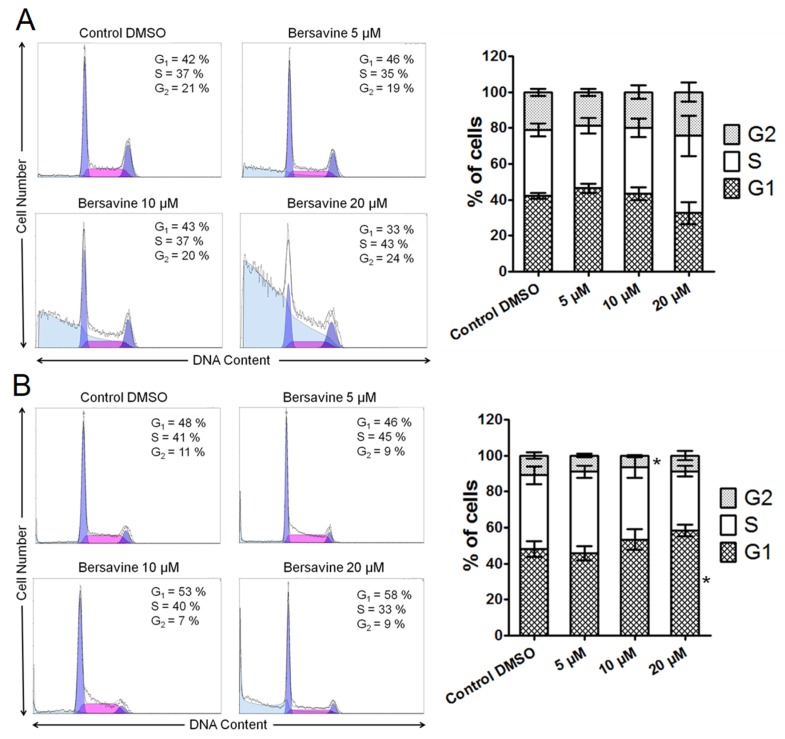
Analysis of the cell cycle after treatment with bersavine. The figure shows representative histograms of Jurkat (**A**) and MOLT-4 (**B**) leukemic cells at a 24 h interval with a mean percentage of cells cycling through phases G1, S, and G2 from a flow cytometry measurement of three separate treatments. The bar graph summarizes cumulative data on the percentage of cells in each phase of the cell cycle. Data are presented as mean values ± SD, *n* = 3. * Significantly different to control (*p* ≤ 0.05).

To understand the antiproliferative effect of bersavine we evaluated the impact of this agent on the distribution of cell populations in various phases of the cell cycle using flow cytometry. After the application of lower concentrations of bersavine on Jurkat cells for 24 h (Figure 4A), we did not see any radical changes compared with negative controls. The sub-G1 population, which was observed in the histogram of Jurkat cells after bersavine treatment in cell debris, was increased with increased concentration of bersavine and is associated with death of cells. Conversely, a significantly (*p* ≤ 0.05) decreased percentage of cells in the G2/M phase of the cell cycle were observed in response to 10 μM of bersavine in MOLT-4 cells with a concomitant slight increase in the amount of G1-phase cells. The application of 20 μM bersavine led to a significantly (*p* ≤ 0.05) increased cell population of MOLT-4 cells in the G1 phase, concomitant with a decrease in the S and G2/M phases of the cell cycle (Figure 4B).

### 2.4. The Effects of Bersavine on the Apoptosis of Jurkat and MOLT-4 Cells

**Figure 5:**
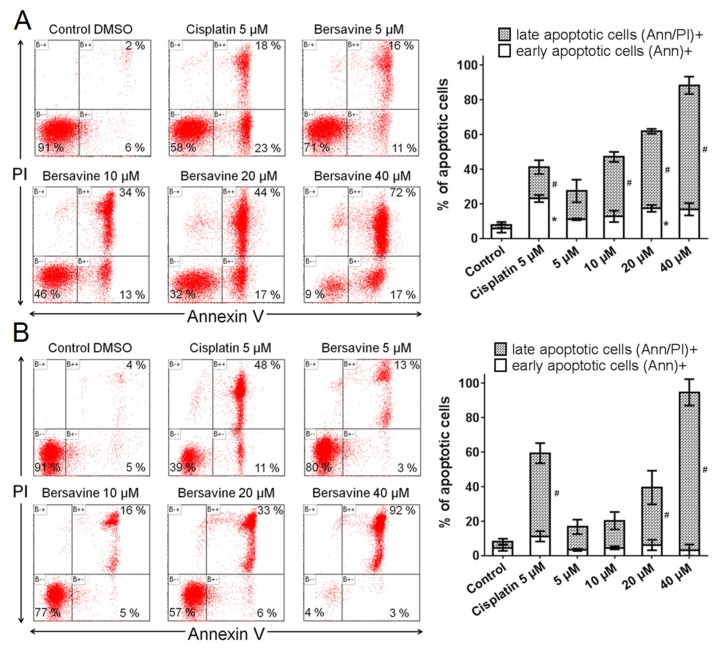
The effect of bersavine on the induction of apoptosis. Apoptosis was determined by Annexin V and propidium iodide (PI) staining 24 h after treatment of Jurkat (**A**) and MOLT-4 (**B**) leukemic cells. Representative histograms of one of three independent measurements are shown. Cells treated with 5 µM of cisplatin were used as a positive control. The bar graph represents the percentage of early and late apoptotic cells detected by flow cytometry (mean ± SD, *n* = 3). * Significantly different to control for early (Annexin V+) apoptotic cells (*p* ≤ 0.05). # Significantly different to control for late (Annexin V/PI+) apoptotic cells (*p* ≤ 0.05).

In view of the decreased amount of viable (Trypan blue nonstaining, i.e., membrane-intact) Jurkat and MOLT-4 cells upon treatment with bersavine, we used flow cytometry assay to analyse whether the cells underwent apoptosis. In this assay, based on the binding of fluorochrome-conjugated Annexin V to phosphatidylserine and propidium iodide to DNA in the presence of Ca^2+^, we identified cells that were alive in the early stage of apoptosis, at the end of apoptosis, or cells in necrosis. Twenty-four hours after the application of 0, 5, 10, 20 and 40 μM of bersavine, the early apoptotic rates were 6%, 11%, 13%, 17% and 17% for Jurkat cells and 5%, 3%, 5%, 6% and 3% for MOLT-4 cells, respectively. The late apoptotic rates were 2%, 16%, 34%, 44% and 72% for Jurkat cells (Figure 5A) and 4%, 13%, 16%, 33% and 92% for MOLT-4 cells (Figure 5B). These experiments indicate that bersavine promotes apoptosis in a dose-dependent fashion, showing an overall higher late apoptotic cell rate compared to the early apoptotic cell rate in both determined cell lines. 

**Figure 6:**
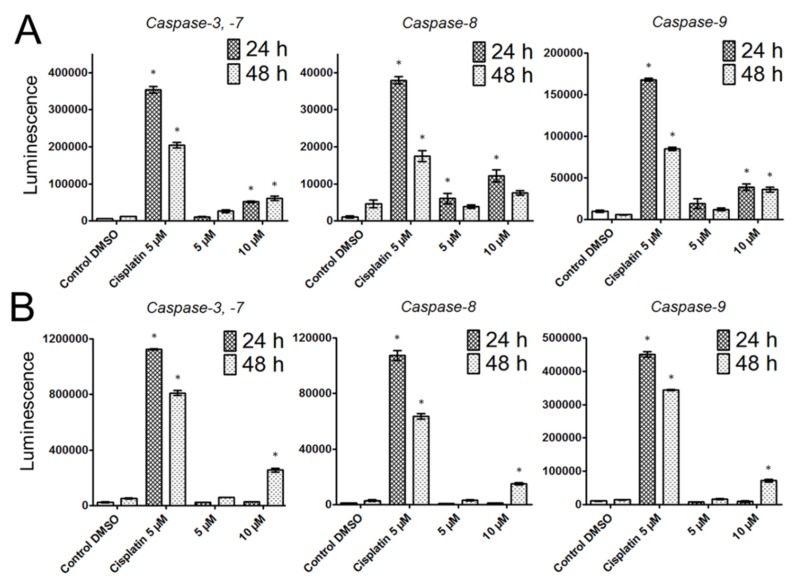
The effect of bersavine on the activity of caspases using luminescence-based assays. The activity of caspases-3/7, caspase-8 and caspase-9 were determined in Jurkat (**A**) and MOLT-4 (**B**) cells 24 and 48 h after treatment. Caspase activities are represented as luminescence intensity. Data are presented as mean values ± SD, *n* = 3. * Significantly different to control (*p* ≤ 0.05).

Subsequently, we evaluated the activation of caspases-3/-7, -8 and -9 to better understand the apoptosis-inducing effect of bersavine. Caspases-3,-7 are the effector caspases of cell death, which are activated by initiating caspase-8 in the case of a death-receptor pathway and -9 in the case of a mitochondrial pathway. Significantly increased levels of caspase-3,-7 were observed after 24 and 48 h following the application of 10 μM of bersavine in Jurkat cells. Initiator caspase-8 was significantly increased compared to controls after 24 h of treatment with bersavine at a concentration of 5 and 10 μM in Jurkat cells, but initiator caspase-9 was increased only after exposure to 10 μM of bersavine after both time courses of treatment (Figure 6A). Contrary to Jurkat cells, MOLT-4 cells showed significantly higher levels of all examined caspases only after 48 h of treatment with 10 μM of bersavine (Figure 6B).

### 2.5. The Effects of Bersavine on the Activation of Checkpoint, Apoptotic and MAPK Signalling Pathways in Jurkat and MOLT-4 Cells

**Figure 7:**
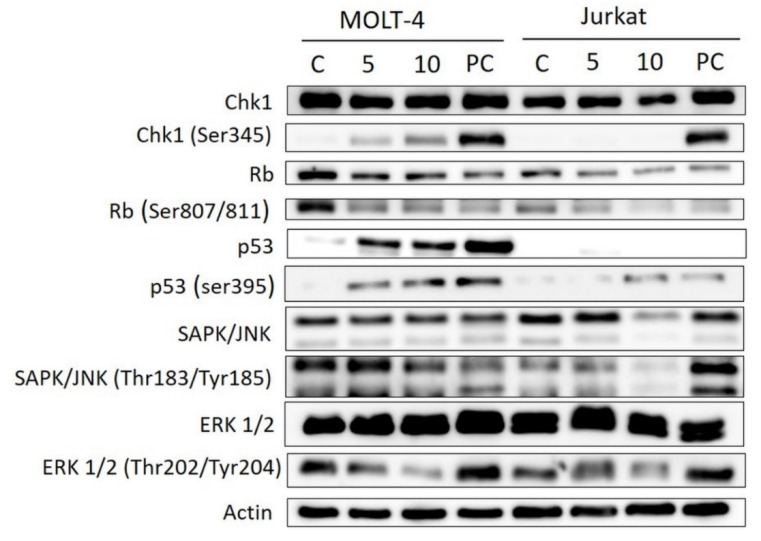
Western blot analysis of proteins that regulate checkpoint responses or induction of cell death in MOLT-4 and Jurkat leukemic cells upon treatment with bersavine. MOLT-4 and Jurkat cells were treated with bersavine for 24 h. Control cells were mock treated with 0.1% DMSO (DMSO) and 5 μM of cisplatin-treated cells were used as a positive control (PC). These experiments were performed at least three times with similar results and a cropped blot is shown from one representative experiment.

Thanks to the Western blotting method and electrophoresis, we investigated the protein expression levels and the activation of cell cycle regulatory checkpoint kinase 1 (Chk1), retinoblastoma tumor suppressor protein (Rb), p53 protein, stress-activated protein kinase/Jun-amino-terminal kinase SAPK/JNK and extracellular regulated kinase (ERK1/2). Bersavine caused activation of Chk1 through phosphorylation at Ser345, accumulation and activation of p53, activation of the Rb protein, a decrease in SAPK/JNK activated through phosphorylation at Thr183/Tyr185 and a decrease in ERK1/2 activated through phosphorylation at Ser202/204 (pERK1/2) in p53-wild-type (p53+) MOLT-4 cells. In Jurkat cells, the activation of the Rb protein and a decrease of pERK1/2 and pSAPK/JNK were detected (Figure 7).

## 3. Discussion

This work is the first report focused on exploring the antiproliferative, cytotoxic and apoptosis-inducing effects of bersavine. In previous studies, the structurally related alkaloid berbamine was clearly confirmed as a promising anticancer drug candidate, which was supported by experimental evidence of strong antiproliferative, cytotoxic and proapoptotic activity [6,7,8,12]. Moreover, berbamine is well-known in Chinese medicine and it has been used as a treatment option for decades [11]. On the other hand, nothing so far is known about the antiproliferative, cytotoxic and apoptosis-inducing effect of bersavine.

In the study of Wang et al., 24 h of treatment with berbamine negatively affected HepG2 hepatocellular cancer cells, resulting in a decrease in cell viability and proliferation in a dose-dependent manner, with an IC_50_ value of 34.5 ± 0.5 µM [11]. The higher impact of berbamine treatment was observed in work oriented on glioma cancer, where the U-87 human glioblastoma cell line was used as an experimental model. By analysing U-87 glioblastoma cell proliferation at a dosing interval of 48 h, it was observed that berbamine inhibited the growth of cancer cells in a dose-dependent manner with an IC_50_ value of 9.8 ± 0.6 µM [7]. Berbamine-treated lung carcinoma cells A549 showed a dose-dependent decrease of viability after 48 h of treatment, with an estimated IC_50_ value of 34.63 ± 1.12 µM [15]. Another recent study by Zhang et al. described the suppression of proliferation of colorectal cancer cells HCT116 and SW480 by a 48 h treatment with berbamine. The IC_50_ values determined were 12.3 ± 1.02 µM for the HCT116 cell line and 16.4 ± 0.89 µM for the SW480 cell line, respectively [14]. For the first time, our study showed that a new *Berberis vulgaris* active constituent bersavine applied for 48 h at 5 μM inhibited both the proliferation and the viability of human leukemic Jurkat and MOLT-4 cells in vitro. The coassayed IC_50_ values of bersavine for the most sensitive human cancer cells in the initial cytotoxicity screen ranged between 8.1 ± 1.7 and 11.0 ± 1.2 µM. In addition, a real-time cell adhesion, proliferation and cytotoxicity xCELLigence system confirmed the high antiproliferative activity of bersavine against cancer cell lines derived from a lung (A549), ovarian (A2780), breast (MCF-7) and hepatocellular (HepG2) cancer during 72 h of continuous monitoring. These results suggest that bersavine has a similar cytotoxic and antiproliferative effect to berbamine, as has been described in the aforementioned studies. 

Mechanism studies showed that berbamine exerts its cytostatic and cytotoxic potency against cancer cells by perturbing the cell cycle and inducing apoptosis. Since the chemical structure of berbamine is closely related to bersavine, it is interesting to compare these observations to our results. According to Zhang et al., berbamine treatment at 20 µg/mL (≈33 µM) for 48 h not only significantly increased the percentage of apoptotic cells in both HCT116 and SW480 cells, but also significantly increased the cell population at the G0/G1 phase and decreased the cell population at the G2/M phase in both cell lines [14]. Also, bersavine in our experiments showed similar effects on MOLT-4 cells because the concentration of 10 µM of bersavine significantly decreased the percentage of leukemic cells in the G2/M phase and 20 µM of treatment significantly increased the percentage of cells in the G1 phase. In another study, berbamine applied to SMMC7721 hepatocellular carcinoma cells caused cell cycle arrest in the G0/G1 phase, induced a loss of mitochondrial membrane potential (Δ*ψ*m) and induced the activation of caspase-3 and caspase-9 [16]. Moreover, the authors provided experimental evidence for the involvement of caspases in apoptosis triggered by berbamine, since the berbamine-induced apoptosis could be blocked by pretreatment with the broad caspase inhibitor Z-VAD-FMK [16]. Berbamine also effectively inhibits the growth of RPMI 8226 human multiple myeloma cells, which is associated with the activation of the GADD45/JNK signalling pathway, apoptosis and upregulation of p53, p21 and GADD45gamma mRNA [17]. On the other hand, Wang et al. described the proapoptotic effects of berbamine, which upregulated the FasL expression in HepG2 cells, activated caspase-8, induced a loss of mitochondrial membrane potential (Δ*ψ*m), with subsequent activation of caspase-9 and -3, which ultimately resulted in the disassembly of HepG2 cells [11]. This study suggests that berbamine induces apoptosis in HepG2 cells through a type II Fas signalling pathway [11]. 

Since previous studies have provided clear evidence that berbamine treatment increased apoptotic rates, we decided to ascertain whether apoptosis also occurs after bersavine treatment. Exposure to bersavine resulted in increased levels of caspase-3/-7, caspase-8 and caspase-9 in Jurkat cells, which was generally more pronounced with a higher concentration of bersavine and in the early period of 24 h of treatment. The apoptotic effect of bersavine in Jurkat cells was considerably higher after 24 h of bersavine treatment by determining caspase activity than in MOLT-4 cells. The same bersavine treatment period caused a higher increase in the percentage of early apoptotic Annexin-V single-positive Jurkat cells assayed by flow cytometry compared to those seen in the MOLT-4 cells. The lack of evidence for increased caspase activity after 24 h of bersavine treatment in MOLT-4 cells can be attributed largely to the pronounced cell cycle arrest of MOLT-4 cells in the G1/S phase at this short incubation time period. Furthermore, considering the increased level of Chk1 phosphorylated at Ser345 and the decreased level of Rb phosphorylated at Ser807/811 after 24 h of bersavine (10 µM) treatment and the significantly increased levels of caspase-3/-7, -8 and -9 after 48 h of treatment, we hypothesise that the early treatment interval action (24 h) of bersavine on MOLT-4 cells primarily lies in arresting the cell-cycle progression rather than apoptosis induction. Our observations also indicate that the proapoptotic effect of bersavine is at least partially mediated via the upregulation of the protein levels of phosphorylated p53 at Ser392 in the case of p53-wild-type (p53+) MOLT-4 cells, similar to berbamine, as was described in work of Zhang et al. [14]. 

## 4. Materials and Methods

### 4.1. Cell Culture and Culture Conditions

The selected human tumour cell lines Jurkat (acute T cell leukemia), MOLT-4 (acute lymphoblastic leukaemia), A549 (lung carcinoma), HT-29 (colorectal adenocarcinoma), PANC-1 (pancreas epithelioid carcinoma), A2780 (ovarian carcinoma), HeLa (cervix adenocarcinoma), MCF-7 (breast adenocarcinoma), SAOS-2 (osteosarcoma) and HepG2 (hepatocellular carcinoma) were purchased from either ATCC (Manassas, VA, USA) or Sigma-Aldrich (St. Louis, MO, USA) and were cultured in accordance with the provider´s culture method guidelines. The cell cultures were maintained under standard cell culture conditions at 37 °C in a humidified incubator in an atmosphere of 5% CO_2_, 95% air. Cells were passaged every 2–3 days to obtain exponential growth. Cells in the maximum range of 20 passages and in an exponential growth phase were used for this study.

### 4.2. Cell Treatment

Bersavine and berbamine—fresh stock solutions of bersavine and berbamine in concentrations of 50 mM were dissolved in dimethyl sulfoxide (DMSO) (Sigma-Aldrich, St. Louis, MO, USA). Stock solutions were freshly prepared before use in the experiments. Bersavine and berbamine were isolated from an alkaloidal extract of the root bark of *Berberis vulgaris* (Berberidaceae). The detailed isolation and identification of the structures (1D-, 2D-NMR, HRMS and optical rotation) of the alkaloid has been published by Hostalkova and colleagues [5]. For the experiments, the stock solutions were diluted using the complete culture medium to create final concentrations of 1–50 μM, making sure the concentration of DMSO was <0.1% to avoid any toxic effects on the cells. Cisplatin and doxorubicin were purchased from Sigma-Aldrich (Sigma-Aldrich, St. Louis, MO, USA). Control cells were sham-treated with a DMSO vehicle only (0.1%; control). Cells treated with 5% DMSO, cisplatin at 5 µM or doxorubicin at 1 µM were used as a positive control.

### 4.3. WST-1 Assay and Growth Percent Calculation

To determine cell proliferation, cell viability, and the cytotoxicity of cells treated with bersavine and berbamine at a single dose of 10 µM or in a broad concentration range of 0.1–100 µM (determination of IC_50_ values), we used a standard colorimetric method measuring a WST-1 tetrazolium salt reduction via mitochondrial dehydrogenase activity. At the onset of the experiments, each cell line was seeded at a previously established optimal density (1 × 10^3^ to 50 × 10^3^ cells per well) in a 96-well plate (TPP, Trasadingen, Switzerland) and the cells were allowed to settle overnight. The cells were treated for 48 h with bersavine. Doxorubicin (Sigma-Aldrich, St. Louis, MO, USA) at a concentration of 1 µM was used as a positive control. At the end of the cultivation period, a WST-1 proliferation and viability assay (Roche, Basel, Switzerland) was performed in accordance with the manufacturer’s instructions. Absorbance was measured using a Tecan Infinite M200 (Tecan, Männedorf, Switzerland) at 440 nm. Each value is the mean of three independent experiments and represents the percentage of proliferation/viability of control, nontreated cells (100%). The growth percent (GP) value was calculated for alkaloid tested. GP represents the mean of the proliferation/viability decrease as a percentage in regard to all of the 9 cell lines treated with bersavine or berbamine. 

### 4.4. Screening for Antiproliferative Activity Using the xCELLigence System

The xCELLigence system (Roche, Basel, Switzerland and ACEA Biosciences, San Diego, CA, USA) was used to monitor cell adhesion, proliferation and cytotoxicity. It was connected and tested by a Resistor Plate before the RTCA Single Plate station was placed inside the incubator at 37 °C and 5% CO_2_. First, the optimal seeding concentration for the experiments was optimized for each cell line. After seeding, the respective number of cells in 190 µL of medium per well of the E-plate 96, the proliferation, attachment and spreading of the cells were monitored every 30 min by the xCELLigence system. Approximately 24 h after seeding, when the cells were in the log growth phase, the cells were exposed in triplicate to 10 µL of sterile deionized water containing bersavine, to obtain final concentrations of 1–50 μM. Controls received sterile deionized water + DMSO with a final concentration of 0.1%. Cells treated with 5% DMSO were used as a positive control. Growth curves were normalized to the time point of treatment. Evaluations were performed using xCELLigence 1.2.1 software (Roche, Basel, Switzerland and ACEA Biosciences, San Diego, CA, USA).

### 4.5. Trypan Blue Exclusion Test for Cell Proliferation and Viability

Cell proliferation and the viability of Jurkat and MOLT-4 cells were determined 24 and 48 h after treatment with 5, 10 and 20 μM of bersavine. Cells treated with 5 µM of cisplatin were used as a positive control. Cell membrane integrity was determined using the Trypan blue exclusion technique—mixing 10 μL of 0.4% Trypan blue (Sigma-Aldrich, St. Louis, MO, USA) and 10 μL of cell suspension. Cell counts were carried out using a Bürker chamber and a Nikon Eclipse E200 light microscope (Nikon, Tokyo, Japan).

### 4.6. Cell Cycle Distribution and Internucleosomal DNA Fragmentation Analysis

Where cell cycle distribution analysis is concerned, the cells were washed with ice-cold Phosphate Buffered Saline PBS and fixed with 70% ethanol. In order to detect low-molecular-weight fragments of DNA, the cells were incubated for 5 min at room temperature in a buffer (192 mL 0.2 M Na_2_HPO_4_ + 8 mL of 0.1 M citric acid, pH 7.8) and then labelled with propidium iodide in Vindelov’s solution for 1 h at 37 °C. The DNA content was determined using a CyAn flow cytometer (Beckman Coulter, Miami, FL, USA) with an excitation wavelength of 488 nm. The data were analysed using Multicycle AV software (Phoenix Flow Systems, San Diego, CA, USA).

### 4.7. Activity of Caspases

The induction of programmed cell death was determined by monitoring the activities of caspases-3/7, caspase-8 and caspase-9 by Caspase-Glo Assays (Promega, Madison, WI, USA) 24 and 48 h after treatment with 5 and 10 μM of bersavine. Cells treated with 5 µM of cisplatin were used as a positive control. The assay provides a proluminogenic substrate in an optimized buffer system. The addition of a Caspase-Glo Reagent results in cell lysis, followed by caspase cleavage of the substrate and the generation of a luminescent signal. A total of 1 × 10^4^ cells were seeded per well using a 96-well plate format (Sigma-Aldrich, St. Louis, MO, USA). After treatment, the Caspase-Glo Assay Reagent was added to each well (50 μL/well) and incubated for 30 min before luminescence was measured using a Tecan Infinite M200 microplate reader (Tecan Group, Männedorf, Switzerland).

### 4.8. Analysis of Apoptosis

Apoptosis was determined by flow cytometry using an Alexa Fluor^®^ 488 Annexin V/Dead Cell Apoptosis kit (Life Technologies, Grand Island, NY, USA) in accordance with the manufacturer’s instructions. The Alexa Fluor^®^ 488 Annexin V/Dead Cell Apoptosis kit employs the property of Alexa Fluor^®^ 488 conjugated to Annexin V to bind to phosphatidylserine in the presence of Ca^2+^, and the property of propidium iodide (PI) to enter cells with damaged cell membranes and to bind to DNA. Measurement was performed immediately using a CyAn flow cytometer (Beckman Coulter, Miami, FL, USA). Listmode data were analysed using Kaluza Analysis 1.3 software (Beckman Coulter, Miami, FL, USA).

### 4.9. Western Blot Analysis

Whole-cell lysates (Cell Lysis Buffer, Cell Signaling Technology, Danvers, MA, USA) were prepared 24 h following treatment of Jurkat and MOLT-4 cells with 5 and 10 μM of bersavine. Cells treated with 0.1% DMSO were used as a negative control. Cells treated with 5 μM of cisplatin were used as a positive control. Quantification of the protein content was performed using the bicinchoninic acid (BCA) assay (Sigma-Aldrich, St. Louis, MO, USA). The lysates (20 µg of purified protein) were loaded into lanes of polyacrylamide gel. After electrophoresis separation, the proteins were transferred to a polyvinylidene difluoride (PVDF) membrane (Bio-Rad, Hercules, CA, USA). Any nonspecific binding of the membranes were blocked for 1 h in a Tris-buffered saline (TBS) containing 0.05% Tween 20 and 10% w/v nonfat dry milk. The membranes were washed in TBS. Incubation with a primary antibody against specific antigens (Chk1, Chk1_serine 345-Cell Signalling, Danvers, MA, USA; Rb, Rb_serine 807 and serine 811–Cell Signalling, Danvers, MA, USA; SAPK/JNK, SAPK/JNK_threonine 183 and tyrosine 185–Cell Signalling, Danvers, MA, USA; β-actin–Sigma-Aldrich, St. Louis, MO, USA; ERK1/2_threonine 202 and tyrosine 204–Cell Signalling, Danvers, MA, USA; p53, p53_serine 392–Exbio, Prague, Czech Republic) was performed at 4° C overnight. The following day, the membranes were washed 5 times with TBS, each time for 5 min, and once with TBS for 10 min, and then incubated with an appropriate secondary antibody (DakoCytomation, Glostrup, Denmark) for 1 h at room temperature. Band detection was performed using a chemiluminiscence detection kit (Roche, Basel, Switzerland). To ensure equal protein loading, each membrane was reprobed and β-actin was detected.

### 4.10. Calculation of IC_50_ Values

IC_50_ values were calculated on the basis of data obtained from proliferation/viability determined by the use of a WST-1 assay and were processed using GraphPad Prism 7 biostatistics (GraphPad Software, San Diego, CA, USA) software. Drug concentrations were plotted against the percentage of cell proliferation/viability and the IC_50_ values were determined using nonlinear regression.

### 4.11. Statistical Analysis

The descriptive statistics of the results were calculated and the charts were made using either Microsoft Office Excel 2010 (Microsoft, Redmond, WA, USA) or GraphPad Prism 7 biostatistics (GraphPad Software, San Diego, CA, USA) software. In this study, all the values were expressed as arithmetic means with the SD of triplicates, unless otherwise noted. For quantitative data, normality testing was performed to assess whether parametric or nonparametric tests should be used. For experiments with parametric variables, the significant differences between the groups were analysed using the Student’s t-test and a *p*-value < 0.05 was considered significant.

## 5. Conclusions

Our study showed for the first time that bersavine, a new bisbenzylisoquinoline alkaloid recently isolated from the *Berberis vulgaris*, displayed a potent inhibitory activity against multiple cancer histotypes. Follow-up mechanistic experiments with human leukemic cells suggest that this new plant alkaloid decreases proliferation by impairing cell cycle progression and induces apoptosis-mediated cell death associated with the upregulation of p53 and activation of caspases-3/-7, -8 and -9. In conclusion, bersavine demonstrated a promising potential with regard to antiproliferative, cytotoxic and proapoptotic activity against cancer cells, which warrants further investigation towards possible translation into clinical therapy.

## Figures and Tables

**Table 1:** Cytotoxicity of bersavine and berbamine following single-dose exposure at a concentration of 10 μM. Doxorubicin at 1 µM was used as a reference drug. Data are shown as mean values ± SD of at least three independent experiments and are expressed as the percent of proliferation of 0.1% dimethyl sulfoxide (DMSO) mock-treated control cells (100%).

Cell Type	Bersavine	Berbamine	Doxorubicin
Jurkat	49 ± 2	53 ± 4	0 ± 3
MOLT-4	27 ± 9	59 ± 15	0 ± 1
A549	97 ± 7	120 ± 10	66 ± 16
HT-29	47 ± 8	53 ± 11	77 ± 12
PANC-1	94 ± 5	116 ± 9	59 ± 9
A2780	77 ± 7	70 ± 4	5 ± 1
HeLa	43 ± 6	21 ± 4	7 ± 10
MCF-7	43 ± 10	43 ± 9	41 ± 7
SAOS-2	113 ± 4	127 ± 6	73 ± 8

**Table 2:** Sensitivity to the antiproliferative activities of bersavine and berbamine following single-dose exposure at a concentration of 10 µM. Doxorubicin at 1 µM was used as a reference drug ^a,b^.

Compound	Mean GP	Range of GP	Most Sensitive Cell Lines	% Cell Growth
Bersavine	66	27–113	MOLT-4, HeLa, MCF-7	27, 43, 43
Berbamine	74	21–127	HeLa, MCF-7, Jurkat	21, 43, 53
Doxorubicin	37	0–77	Jurkat, MOLT-4, A2780	0, 0, 5

^a^ Mean growth percent (GP) value was calculated for each compound as an average of 9 cell lines’ proliferation as a percentage. ^b^ Range of growth percentage, as well as the three most sensitive cell lines, with growth percentage values indicated for each compound.

**Table 3:** IC_50_ values of bersavine and berbamine in human cancer cells ^a,b^.

Cell Type	Bersavine	Berbamine
Jurkat	9.9 ± 0.3	10.0 ± 0.5
MOLT-4	10.3 ± 2.1	16.4 ± 0.8
HT-29	8.1 ± 1.7	6.1 ± 1.3
HeLa	11.0 ± 1.2	8.3 ± 0.7
MCF-7	9.7 ± 0.4	10.7 ± 0.8

^a^ Results are expressed in µM. ^b^ Results are the mean values ± standard deviations of at least three independent replications.

## Supplementary Materials

The following are available online at https://www.mdpi.com/1420-3049/25/4/964/s1, Figure S1: IC_50_ curves of bersavine for the most sensitive cancer cell lines.

## Abbreviations

1D-NMR	one-dimensional nuclear magnetic resonance;
2D-NMR	two-dimensional nuclear magnetic resonance;
BCA	bicinchoninic acid;
Chk1	Checkpoint kinase 1
DMSO	dimethyl sulfoxide
DNA	doxyribonucleic acid
ERK1/2	extracellular signal-regulated kinase
FasL	Fas ligand
GADD45	growth arrest and DNA damage 45 proteins
JNK	c-Jun N-terminal kinase
GP	growth percent
*h*AChE	human acetylcholinesterase
*h*BuChE	humanbutyrylcholinesterase
HRMS	high resolution mass spectrometry
IC_50_	half maximal inhibitory concentration
MAPK	mitogen-activated protein kinase
NF-κB	nuclear factor-kappaB
p53	tumor suppressor protein p53
PI	propidium iodide
POP	prolyl oligopeptidase
PVDF	polyvinylidene difluoride
ROS	reactive oxygen species
Akt	protein kinase B
RTCA	real-time cell analysis
SD	standard deviation
TBS	Tris-buffered saline.
