# Supplementary material for: Bersavine: A Novel Bisbenzylisoquinoline Alkaloid with Cytotoxic, Antiproliferative and Apoptosis-Inducing Effects on Human Leukemic Cells
Source: Molecules. 2020 Feb 20;25(4):964. doi: 10.3390/molecules25040964 (PMC7071104; doi:10.3390/molecules25040964)
Supplement: Supplementary file 1 [file molecules-25-00964-s001.pdf]

Supplementary data

Bersavine: a novel bisbenzylisoquinoline alkaloid with cytotoxic, antiproliferative and apoptosis-inducing effect on human leukemic cells

Darja Koutova[a], Monika Kulhava[a], Radim Havelek[a*], Martina Majorosova[a], Karel Kralovec[b], Klara Habartova[a], Anna Hostalkova[c], Lubomir Opletal[c], Lucie Cahlikova[c], Martina Rezacova[a]

[a]Department of Medical Biochemistry, Faculty of Medicine in Hradec Kralove, Charles University, Simkova 870, Hradec Kralove 500 03, Czech Republic

[b]Department of Biological and Biochemical Sciences, Faculty of Chemical Technology, University of Pardubice, Studentska 573, Pardubice 532 10, Czech Republic

[c]ADINACO Research Group, Department of Pharmaceutical Botany, Faculty of Pharmacy, Charles University, Heyrovskeho 1203, Hradec Kralove 500 05, Czech Republic

**Corresponding author*

Radim Havelek, Ph.D.
Department of Medical Biochemistry,
Faculty of Medicine in Hradec Králové,
Charles University,
Šimkova 870,
500 03 Hradec Králové,
Czech Republic,
Tel.: +420495816293,
E-mail address: havelekr@lfhk.cuni.cz

IC_{50} curves of bersavine for the most sensitive cancer cell lines (cell lines demonstrating at least 50% inhibition of proliferation at 10 µM single-dose treatment)

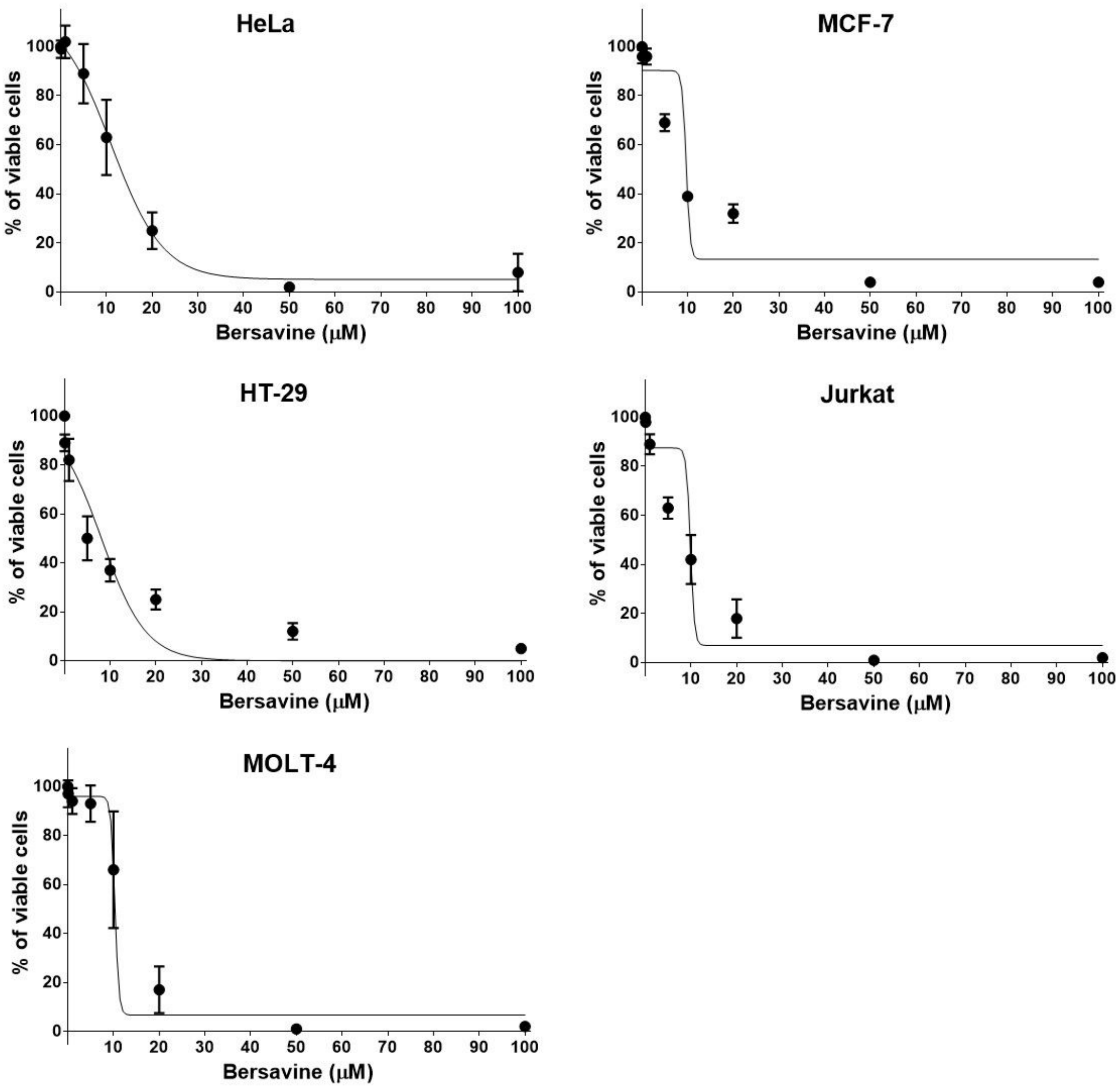

Fig. S1. IC_{50} curves of bersavine for the most sensitive cancer cell lines HeLa, MCF-7, HT-29, Jurkat and MOLT-4. IC_{50} values are given in the table 3. The results are expressed as mean values of three independent experiments ± SD.
